# Supplementary material for: Role of Percutaneous Endoscopic Gastrostomy for the Nutrition of Head and Neck Cancer Patients before and up to 6 Months after Cancer Treatment
Source: Cancers (Basel). 2024 Sep 12;16(18):3138. doi: 10.3390/cancers16183138 (PMC11430239; doi:10.3390/cancers16183138)
Supplement: Supplementary file 1 [file cancers-16-03138-s001.zip › cancers-3184364-supplementary.pdf]

Supplement tables

# Role of Percutaneous Endoscopic Gastrostomy for the Nutrition of Head and Neck Cancer Patients before and up to 6 Months after Cancer Treatment

**Mussab Kouka [1], Sophie Brand [1], Sven Koscielny [1], Thomas Bitter [1], Klaus Pietschmann [2], Thomas Ernst [3] and Orlando Guntinas-Lichius [1,*]**

**Supplement Table S1.** Symptoms caused by the tumor

| **Parameter** | **Frequency (N)** | **%** |
|---|---|---|
| All | 289 | 100 |
| **Symptoms** | | |
| No | 9 | 3.1 |
| Yes | 280 | 96.9 |
| **Swallowing disorders** | | |
| Yes | 104 | 36.0 |
| No | 185 | 64.0 |
| **Pain during nutrition** | | |
| Yes | 150 | 51.9 |
| No | 139 | 48.1 |
| **Globus pharyngeus** | | |
| Yes | 136 | 47.1 |
| No | 153 | 52.9 |
| **Hoarseness** | | |
| Yes | 68 | 23.5 |
| No | 221 | 76.5 |
| **Coughing** | | |
| Yes | 14 | 4.8 |
| No | 275 | 95.2 |
| **Gag reflex during nutrition** | | |
| Yes | 7 | 2.4 |
| No | 282 | 97.6 |
| **Dyspnea** | | |
| Yes | 37 | 12.8 |
| No | 252 | 87.2 |
| **Taste disorders** | | |
| Yes | 2 | 0.7 |
| No | 287 | 99.3 |
| **Loss of appetite** | | |
| Yes | 11 | 3.8 |
| No | 278 | 96.2 |
| **Edema in the area of the tumor** | | |
| Yes | 159 | 55.0 |
| No | 130 | 45.0 |
| **Number of symptoms** | | |
| 0 | 9 | 3.1 |

| | | |
|---|---|---|
| 1 | 53 | 18.3 |
| 2 | 71 | 24.6 |
| 3 | 65 | 22.5 |
| 4 | 56 | 19.4 |
| 5 | 21 | 7.3 |
| 6 | 10 | 3.5 |
| | **Mean±SD** | **Median, Range** |
| Number of symptoms | 2.79 ± 1.5 | 3, 0 - 7 |

SD= standard deviation.

**Supplement Table S2.** Long-term outcome and follow-up

| **Parameter** | **Frequency (N)** | **%** |
|---|---|---|
| **Long-term complication** | | |
| All | 289 | 100 |
| **Tumor recurrence** | | |
| Yes | 77 | 26.6 |
| No | 135 | 46.7 |
| No recurrence, but progress | 57 | 19.7 |
| Unknown | 20 | 6.9 |
| **Death** | 141 | 22.9 |
| Yes | 114 | 39.4 |
| No or unknown | 175 | 60.6 |
| | **Mean±SD** | **Median, Range** |
| Follow-up, all patients in months | 31.6 ± 39.0 | 16, 0-201 |
| Follow-up, patients alive in days | 32.6 ± 40.6 | 17, 0-201 |
| Duration until recurrence after initial diagnosis in days (N=77) | 441.43 ± 343.64 | 348; 58-1565 |
| Duration from recurrence to death in days (N=31) | 301.19 ± 232.78 | 292; 25-1054 |

SD= standard deviation.

**Supplement Table S3**. Time course of the parameters collected for the follow-up

| | **Before cancer treatment (Z0)** | | **1 week after end of cancer treatment (Z1)** | | **6 weeks after end of cancer treatment (Z2)** | | **6 months after end of cancer treatment (Z3)** | |
|---|---|---|---|---|---|---|---|---|
| **Parameter** | **N** | % | **N** | % | **N** | % | **N** | % |
| **All patients** | 289 | 100.0 | 289 | 100.0 | 289 | 100.0 | 289 | 100 |
| **Died before assessment** | | | | | | | | |
| Yes | 0 | 0.0 | 1 | 0.3 | 14 | 4.8 | 28 | 9.7 |
| No | 289 | 100.0 | 288 | 99.7 | 275 | 95.2 | 261 | 90.3 |
| **No information** | | | | | | | | |
| Yes | 0 | 0.0 | 2 | 0.7 | 20 | 6.9 | 31 | 10.7 |
| No | 289 | 100.0 | 287 | 99.3 | 269 | 93.1 | 258 | 89.3 |
| | | | | | | | | |
| **All patients alive** | **N= 289** | 100.0 | **N= 286** | 100.0 | **N= 255** | 100.0 | **N= 230** | 100.0 |
| **Swallowing disorders** | | | | | | | | |
| Yes | 104 | 36.0 | 122 | 42.7 | 95 | 37.3 | 85 | 37.0 |
| No | 185 | 64.0 | 164 | 57.3 | 160 | 62.7 | 145 | 63.0 |
| **Pain during nutrition** | | | | | | | | |
| Yes | 150 | 51.9 | 151 | 52.8 | 74 | 29.0 | 51 | 22.2 |
| No | 139 | 48.1 | 135 | 47.2 | 181 | 71.0 | 179 | 77.8 |
| **Loss of appetite** | | | | | | | | |
| Yes | 11 | 3.8 | 10 | 3.5 | 31 | 12.2 | 27 | 11.7 |
| No | 278 | 96.2 | 276 | 96.5 | 224 | 87.8 | 203 | 88.3 |
| **Gag reflex during nutrition** | | | | | | | | |
| Yes | 7 | 2.4 | 47 | 16.4 | 22 | 8.6 | 15 | 6.5 |
| No | 282 | 97.6 | 239 | 83.6 | 233 | 91.4 | 215 | 93.5 |
| **Taste disorders** | | | | | | | | |
| Yes | 2 | 0.7 | 3 | 1.0 | 11 | 4.3 | 22 | 9.6 |
| No | 287 | 99.3 | 283 | 99.0 | 244 | 95.7 | 208 | 90.4 |
| **Globus pharyngeus** | | | | | | | | |

| | | | | | | | | |
|---|---|---|---|---|---|---|---|---|
| Yes | 136 | 47.1 | 45 | 15.7 | 16 | 6.3 | 12 | 5.2 |
| No | 153 | 52.9 | 241 | 84.3 | 239 | 93.7 | 218 | 94.8 |
| **Swallowing therapy** | | | | | | | | |
| Yes | 0 | 0 | 51 | 17.8 | 9 | 3.5 | 9 | 3.9 |
| No | 289 | 100 | 235 | 82.2 | 246 | 96.5 | 221 | 96.1 |
| **Oral nutrition** | | | | | | | | |
| Yes | 185 | 64.0 | 187 | 65.4 | 199 | 78.1 | 189 | 82.2 |
| Only porridge/liquid food | 104 | 36.0 | 41 | 14.3 | 21 | 8.2 | 11 | 4.8 |
| No | 0 | 0 | 58 | 20.3 | 35 | 13.7 | 30 | 13.0 |
| **Oral nutrition including porridge/liquid food** | | | | | | | | |
| Yes | 289 | 100 | 228 | 79.7 | 220 | 86.3 | 200 | 87.0 |
| No | 0 | 0 | 58 | 20.3 | 35 | 13.7 | 30 | 13.0 |
| **Oral nutrition excluding liquid food** | | | | | | | | |
| Yes | 185 | 64.0 | 187 | 65.4 | 199 | 78.0 | 189 | 82.2 |
| No | 104 | 36.0 | 99 | 34.6 | 56 | 22.0 | 41 | 17.8 |
| **Oral supplementary food** | | | | | | | | |
| Yes | 0 | 0 | 16 | 5.6 | 23 | 9.0 | 23 | 10.0 |
| No | 289 | 100 | 270 | 94.4 | 232 | 91.0 | 207 | 90.0 |
| **Enteral nutrition** | | | | | | | | |
| Yes | 0 | 0 | 70 | 24.5 | 60 | 23.5 | 53 | 23.0 |
| No | 289 | 100 | 216 | 75.5 | 195 | 76.5 | 177 | 77.0 |
| **Parenteral centralized** | | | | | | | | |
| Yes | 0 | 0 | 1 | 0.3 | 1 | 0.4 | 4 | 1.7 |
| No | 289 | 100 | 285 | 99.7 | 254 | 99.6 | 226 | 98.2 |
| **Parenteral peripheral** | | | | | | | | |
| Yes | 0 | 0 | 4 | 1.4 | 1 | 0.4 | 1 | 0.4 |
| No | 289 | 100 | 282 | 98.6 | 254 | 99.6 | 229 | 99.6 |
| **Tube feeding, total** | | | | | | | | |
| Yes | 0 | 0 | 70 | 24.5 | 61 | 23.9 | 55 | 23.9 |

| | | | | | | | | |
|---|---|---|---|---|---|---|---|---|
| No | 289 | 100 | 216 | 75.5 | 194 | 76.1 | 175 | 76.1 |
| **Port** | | | | | | | | |
| Yes | 0 | 0 | 0 | 0 | 1 | 0.4 | 2 | 0.9 |
| No | 289 | 100 | 286 | 100.0 | 254 | 99.6 | 228 | 99.1 |
| **NG probe** | | | | | | | | |
| Yes | 0 | 0 | 30 | 10.5 | 2 | 0.8 | 0 | 0 |
| No | 289 | 100 | 256 | 89.5 | 253 | 99.2 | 230 | 100 |
| **PEG** | | | | | | | | |
| Yes | 0 | 0 | 40 | 14.0 | 58 | 22.7 | 53 | 23.0 |
| No | 289 | 100 | 246 | 86.0 | 197 | 77.3 | 177 | 77.0 |
| **Restored dental status** | 280 | 100.0 | 279 | 100.0 | 250 | 100.0 | 228 | 100.0 |
| Yes | 197 | 70.4 | 200 | 71.7 | 189 | 75.6 | 172 | 75.4 |
| No | 83 | 29.6 | 79 | 28.3 | 61 | 24.4 | 56 | 24.6 |
| | **Mean±SD** | **Median, Range** | **Mean±SD** | **Median, Range** | **Mean±SD** | **Median, Range** | **Mean±SD** | **Median, Range** |
| Weight in kg | 77.70 ± 17.32 | 77; 41-145 | 76.57 ± 17.18 | 75, 41-144 | 76.08 ± 17.09 | 74, 43-140 | 74.47 ± 16.9 | 71, 44-140 |
| BMI | 25.87 ± 5.09 | 25.5, 15.4-43.3 | 25.45 ± 5.08 | 25, 15.1-43.9 | 25.28 ± 5.08 | 24.9, 14.7-43.9 | 24.72 ± 4.85 | 24.2, 15.0-42.3 |
| Karnofsky performance status in percent | NA | NA | 86.89 ± 12.91 | 90, 20-100 | 85.96 ± 11.28 | 90, 20-100 | 86.43 ± 11.69 | 90, 20-100 |

NG tube= nasogastric tube; PEG= percutaneous endoscopic gastrostomy; SD= standard deviation; BMI= body mass index, port = totally implantable venous access device; NA= not applicable.

**Supplement Table S4**. Normality test for weight, BMI and Karnofsky performance status for the follow-up

| | **Before cancer treatment (Z0)** | | **1 week after end of cancer treatment (Z1)** | | **6 weeks after end of cancer treatment (Z2)** | | **6 months after end of cancer treatment (Z3)** | |
|---|---|---|---|---|---|---|---|---|
| **Parameter** | **PEG, Yes** | **PEG, No** | **PEG, Yes** | **PEG, No** | **PEG, Yes** | **PEG, No** | **PEG, Yes** | **PEG, No** |
| | **p** | **p** | **p** | **p** | **p** | **p** | **p** | **p** |
| Weight in kg | 0.133 | 0.020 | 0.200 | 0.002 | 0.200 | 0.002 | 0.200 | 0.003 |
| BMI | 0.200 | 0.042 | 0.200 | 0.046 | 0.200 | <0.001 | 0.200 | 0.008 |
| Karnofsky performance status | NA | NA | <0.001 | <0.001 | <0.001 | <0.001 | <0.001 | <0.001 |

PEG= percutaneous endoscopic gastrostomy; BMI= body mass index; not significant p-values (p>0.05) in bold.; NA= not applicable.
